# Nanoscale Analysis of SSB–RecO Interplay in SSB Interaction with the Target

**DOI:** 10.3390/ijms27198858

**Published:** 2026-10-04

**Authors:** Shivkumar Sharma Irukuvajjula, Shaun Filliaux, Yuri L. Lyubchenko

**Affiliations:** Department of Pharmaceutical Sciences, University of Nebraska Medical Center, Omaha, NE 68198, USA; shiva.shivkumar.sharma@gmail.com (S.S.I.); shaun.filliaux@unmc.edu (S.F.)

**Keywords:** *E. coli* SSB, *E. coli* RecO, AFM, single molecule

## Abstract

Single-stranded DNA-binding protein (SSB) coats single-stranded DNA (ssDNA) and recruits downstream factors, yet this same coating must be cleared for SSB-interacting proteins to bind. RecA is a central protein in homologous recombination, which requires SSB-free ssDNA to load during the process. RecO, a recombination mediator protein, is thought to regulate this handoff, but it has not been shown directly that RecO drives the release of full-length, ssDNA-bound SSB. Using atomic force microscopy, we show that RecO actively displaces full-length wild-type SSB from a defined ssDNA substrate, reducing occupancy from 67% to 29%. Displacement efficiency decreases after deleting the C-terminal tip of SSB (ΔC8) or after adding a single alanine at the C-terminus of SSB (A179). Molecular dynamics simulations show that A179 retains its native RecO contact but additionally engages a non-native surface, producing a specific, over-engaged complex, suggesting that these interprotein interactions may affect SSB-ssDNA contacts. Together, these findings suggest that RecO-driven SSB clearance is governed by the protein–protein interface rather than by ssDNA-binding affinity alone, thereby acting as a checkpoint against indiscriminate SSB removal during genome maintenance.

## 1. Introduction

Single-stranded DNA-binding protein (SSB) is an indispensable participant in virtually all aspects of DNA metabolism in *Escherichia coli* and across the bacterial kingdom. SSB functions mostly as a stable homo-tetramer [1]. Each subunit contains an N-terminal oligonucleotide/oligosaccharide binding (OB) fold domain that directly binds to the single-stranded DNA (ssDNA) and the C-terminal region of the protein containing an intrinsically disordered linker (IDL) and a highly conserved acidic tip [1,2]. The four OB-fold domains cooperate to wrap ssDNA partially or completely, occluding approximately 35 nucleotides per tetramer in the partial form and 65 nucleotides per tetramer in the fully bound state [3,4,5]. By coating the intermediates that arise during replication, repair, and recombination, SSB protects them from nucleolytic degradation and inhibits secondary structure formation, thereby enabling downstream enzymatic processing to proceed efficiently. Beyond this protective role, SSB has emerged as a master organizational hub of genome maintenance [6]. The C-terminal tip serves as an interaction interface for a large and diverse cohort of SSB-interacting proteins (SIPs), including the chi subunit of DNA polymerase III, PriA helicase, ExoI, RecQ, RecO, and RecG, which are key factors in DNA replication, repair, and recombination [6,7,8,9,10,11]. Through these interactions, SSB actively recruits and coordinates the downstream machinery that processes ssDNA intermediates, hence establishing its role also as a dynamic platform for genomic maintenance [12].

Understanding how SIPs precisely recognize and engage with SSB has been the subject of active investigation.

**Figure 1:**
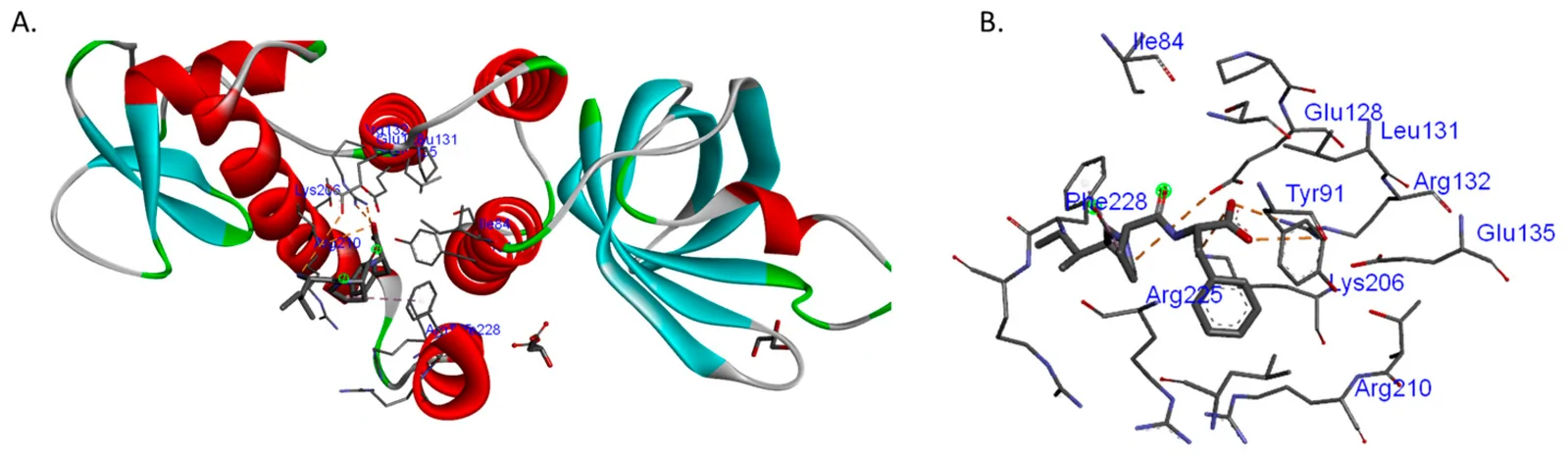
Crystal structure of SSB-Ct bound to RecO, PDB ID: 3Q8D (**A**) SSB-Ct interacting with residues from RecO and (**B**) RecO pocket atoms involved in SSB-Ct binding.

RecO, a member of the SIP family, functions as a regulatory protein that actively reshapes SSB’s engagement with ssDNA. The specific outcome varies with the reaction and the organism [13]. RecO is central to the RecFOR pathway [14,15,16], which relieves the barrier SSB poses to RecA loading during homologous recombination. Biochemical studies for *E. coli* show that RecO and RecR together overcome the SSB’s inhibition of RecA loading [17] and that RecO can form a stable 1:1 complex with SSB, while annealing ssDNA [18,19]. Studies in other organisms revealed diverse regulatory mechanisms [20,21,22,23,24,25]. These studies establish RecO as a factor regulating SSB-ssDNA interactions via allosteric weakening of SSB’s intrinsic affinity [26], competitive eviction [25], and remodeling of the SSB-ssDNA complex into an annealing-competent intermediate [11,19,20]. The crystal structure of *E. coli.* SSB-Ct bound to RecO highlights the role of SSB tip residues in this interaction (Figure 1).

However, biochemical studies cannot distinguish whether SSB is displaced, remains indirectly tethered (as proposed for the *E. coli* system), or persists as a mixture of intermediate states. The evidence for RecO-driven displacement was obtained for *D. radiodurans* [25], whose SSB and RecO architecture differs substantially from that of *E. coli.*

Here, we address SSB–RecO interactions using atomic force microscopy (AFM), enabling us to directly visualize individual protein-DNA complexes and characterize these interactions. Using ssDNA substrate capable of accommodating a single SSB tetramer, we show that RecO actively drives dissociation of full-length, DNA-bound wtSSB, and that this dissociation is gated strictly by the C-terminal acidic tip. The SSB displacement is abolished by either removing the tip entirely (ΔC8) or extending it by a single residue (A179). Together with MD simulations, these findings establish the C-terminal acidic tip as a critical segment of SSB for its interaction with RecO, thereby defining its potential regulatory role in SSB-DNA interactions.

## 2. Results

### 2.1. DNA Substrate

**Figure 2:**
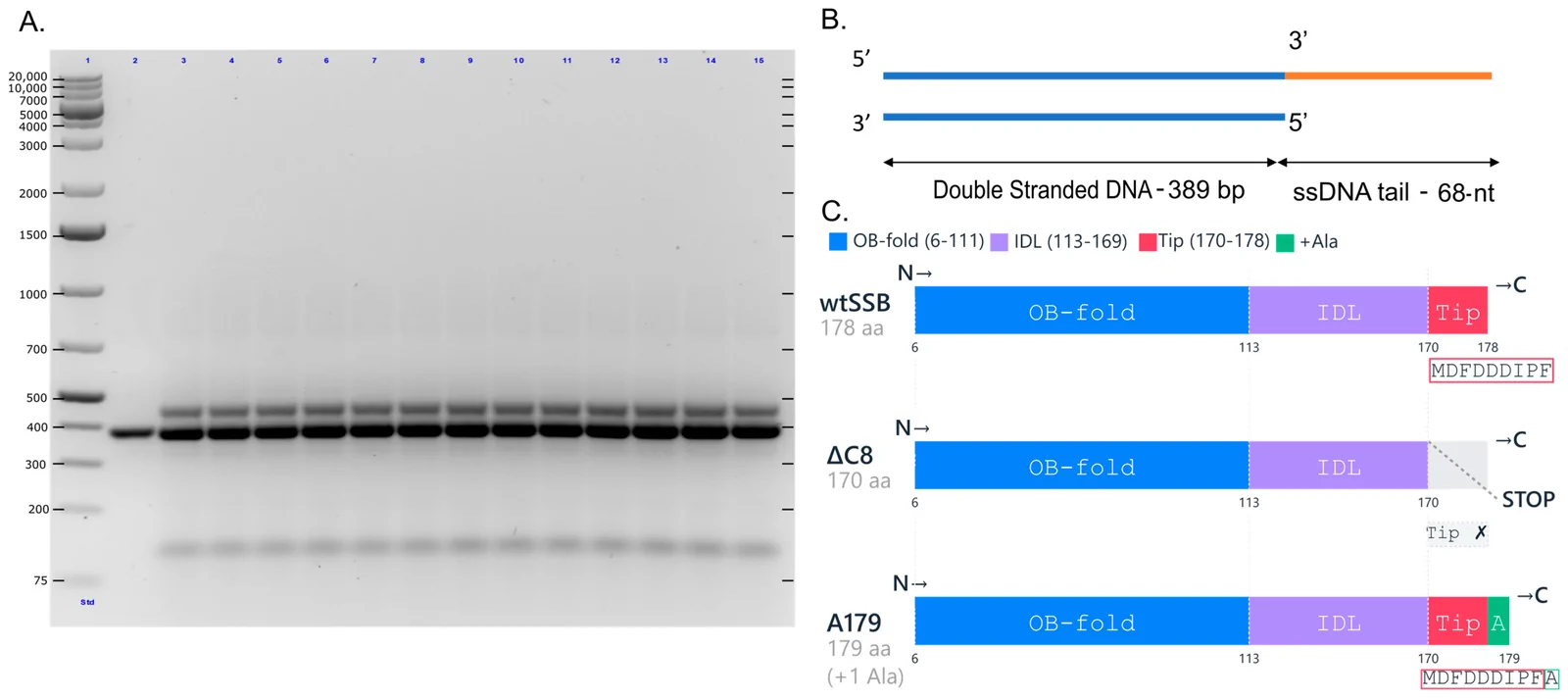
DNA substrate design. (**A**) Agarose gel electrophoresis of the substrate preparation (lane 1, DNA ladder, sizes in bp; lanes 2-15, substrate samples), resolving the ~400-500 bp duplex product. (**B**): Schematic of the DNA substrate—389 bp dsDNA joined to a 68 nt ssDNA tail (91 nt oligo + 20 nt adaptor with 3 nt SapI site). (**C**) Schematic protein domain representation of wtSSB and SSB variants.

In our AFM studies, we used a hybrid DNA substrate in which an ssDNA segment (~68 nucleotides) is attached to the end of the long DNA duplex (~300 bp), allowing direct AFM visualization of complexes of ssDNA with single-stranded DNA-binding proteins [27,28,29,30,31]. The schematic is shown in Figure 2B. The designed DNA substrate was gel-purified as described in the Section 4. Figure 2A shows the gel electrophoresis picture illustrating the difference in migration between naked dsDNA and dsDNA with an attached ssDNA tail.

### 2.2. SSB Variants Used in This Study

To map the structural requirements of the SSB C-terminal domain for RecO–SSB interaction, we used three SSB constructs throughout this study (Figure 2C). Wild-type SSB (wtSSB) carries the complete intrinsically disordered linker (IDL), including all three PXXP motifs, together with the intact eight-residue acidic tip (DFDDDIPF). ΔC8 lacks the eight C-terminal residues that make up the acidic tip. A179 retains the complete IDL and an unaltered native tip sequence, but carries a single alanine appended to the C-terminus after the terminal Phe residue. Together, these variants test the contributions of the C-terminal tip: ΔC8 tests whether an intact IDL is sufficient for RecO–SSB interaction in the absence of the tip, while A179 tests the tip’s sequence specificity.

### 2.3. RecO Binds Weakly to the ssDNA and ΔC8

**Figure A1:**
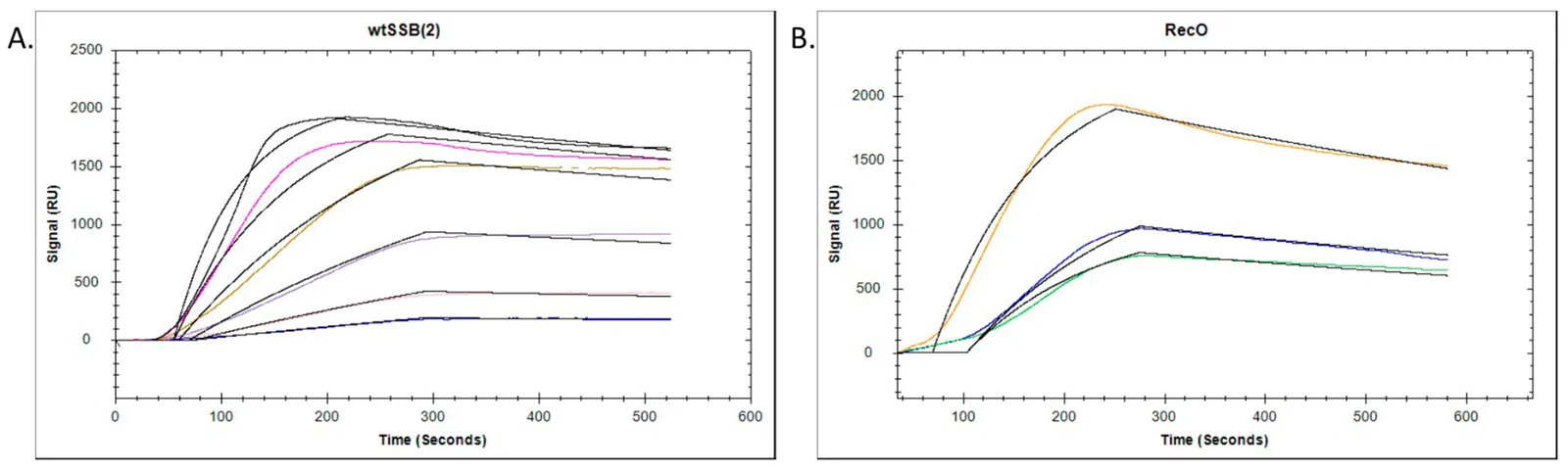
RecO binds the ssDNA oligo used in this study, but more weakly than wtSSB. SPR sensorgrams for (**A**) wtSSB and (**B**) RecO binding to the immobilized oligo across a range of protein concentrations, overlaid with global 1:1 (Langmuir) kinetic fits (black lines). The colored lines are various concentrations of protein used in the titration.

**Figure A2:**
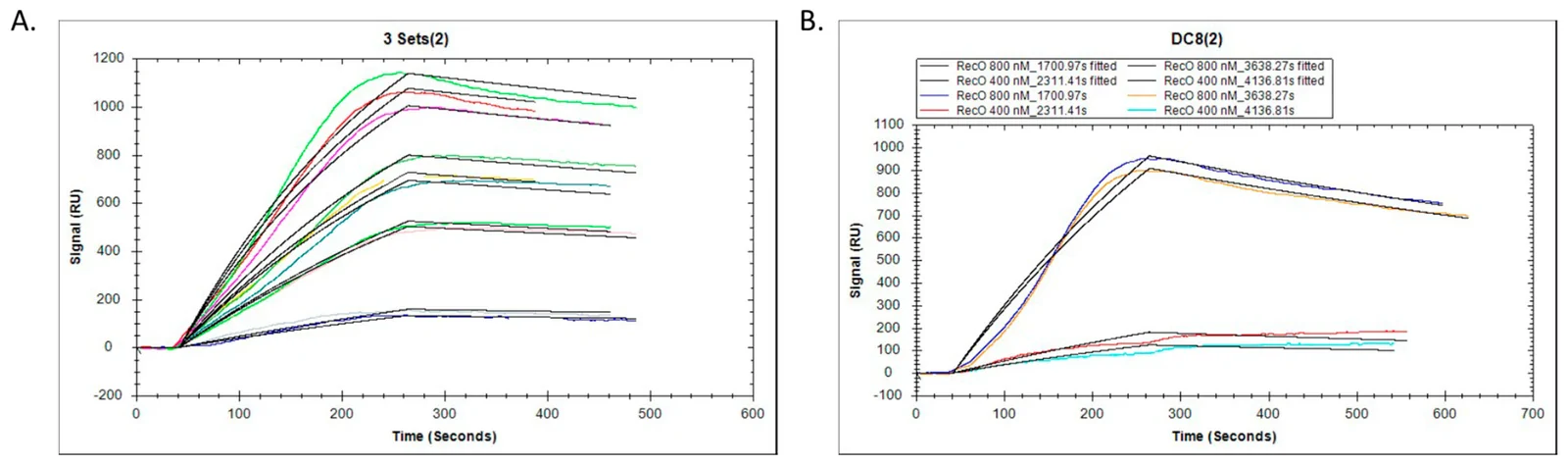
RecO binding to ssDNA immobilized (**A**) wtSSB and (**B**) ΔC8 binding, overlaid with global 1:1 (Langmuir) kinetic fits (black lines).

We used surface plasmon resonance (SPR) to assess the ability of RecO and wtSSB to bind the oligonucleotide used as an ssDNA tail in the study; data were fitted to 1:1 Langmuir kinetic models. wtSSB bound to the oligonucleotide with an estimated K_D_ value of 15 nM [23,32]. Whereas RecO bound with an estimated K_D_ value of ~90 nM, reflecting a lower affinity to the DNA substrate compared to wtSSB. RecO’s tendency to aggregate limited its titration to a narrow concentration range (500–1000 nM), so this value should be regarded as an estimate rather than a precise measurement; nonetheless, this difference in estimated K_D_ indicates that RecO binds the oligo more weakly than wtSSB (Figure A1). RecO titrations on ssDNA-bound wtSSB and ΔC8 were performed as described in Materials and Methods. RecO solubility limitations restricted the data set to three independent injection series for wtSSB and a single injection series for ΔC8. Due to this constraint, a complete kinetic analysis was not feasible. Instead, apparent dissociation constants (K_D_) were estimated using a Langmuir 1:1 binding model. RecO displayed a ~4-fold-higher K_D_ for ΔC8 than for wtSSB, indicating weaker binding of RecO to the truncated SSB variant (Figure A2). SPR experiments with the A179 variant were not encouraged due to the limited solubility of RecO.

### 2.4. Design and Validation of the ssDNA-Tailed AFM Substrate

**Figure 3:**
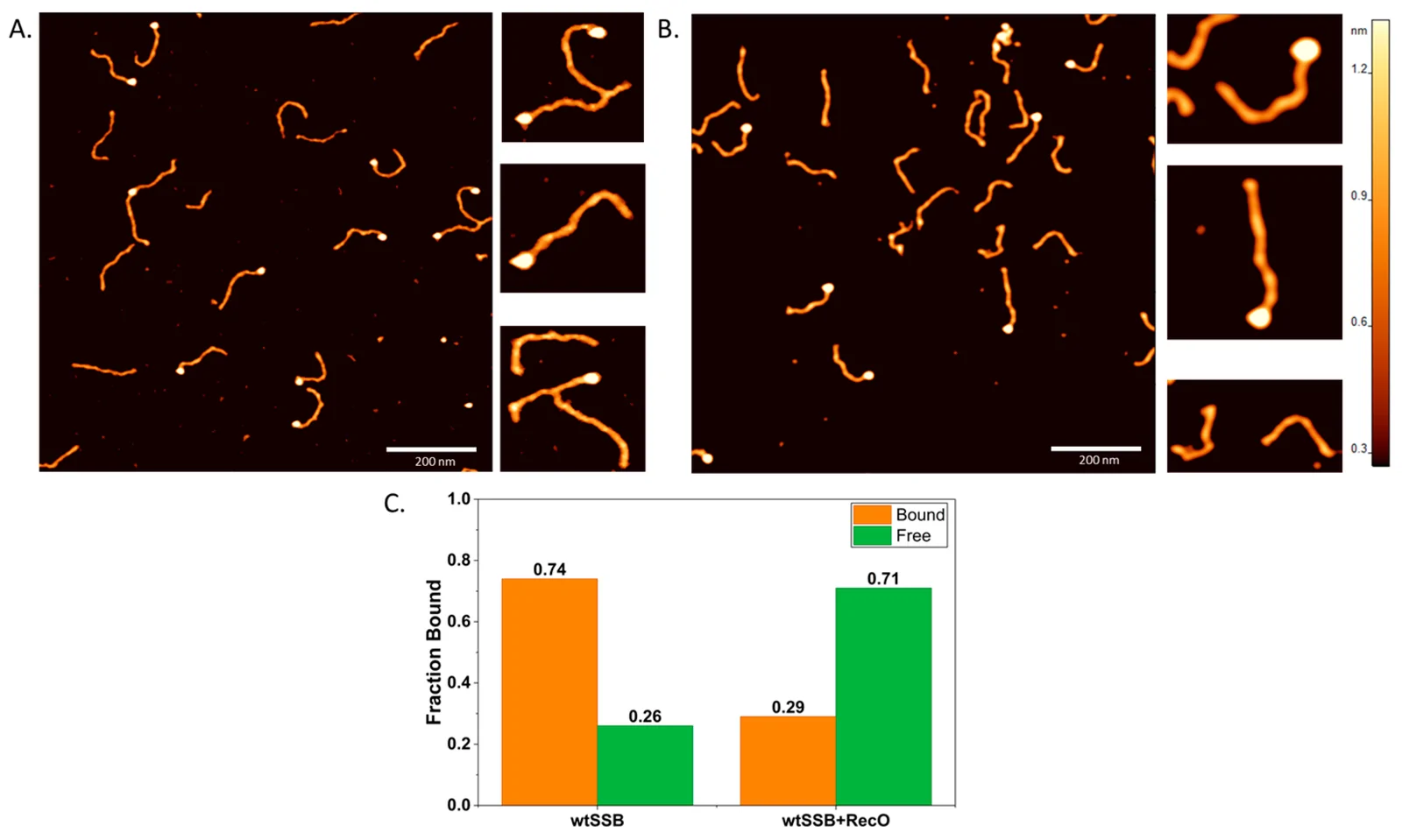
AFM imaging of wtSSB on the DNA substrate ± RecO. (**A**) Representative field and zoomed-in insets, DNA + wtSSB alone. (**B**) Representative field and zoomed-in insets, DNA + wtSSB + RecO. Scale bars: 200 nm. (**C**) Fraction of DNA molecules scored as SSB-bound vs. free, wtSSB alone vs. wtSSB + RecO.

**Figure 4:**
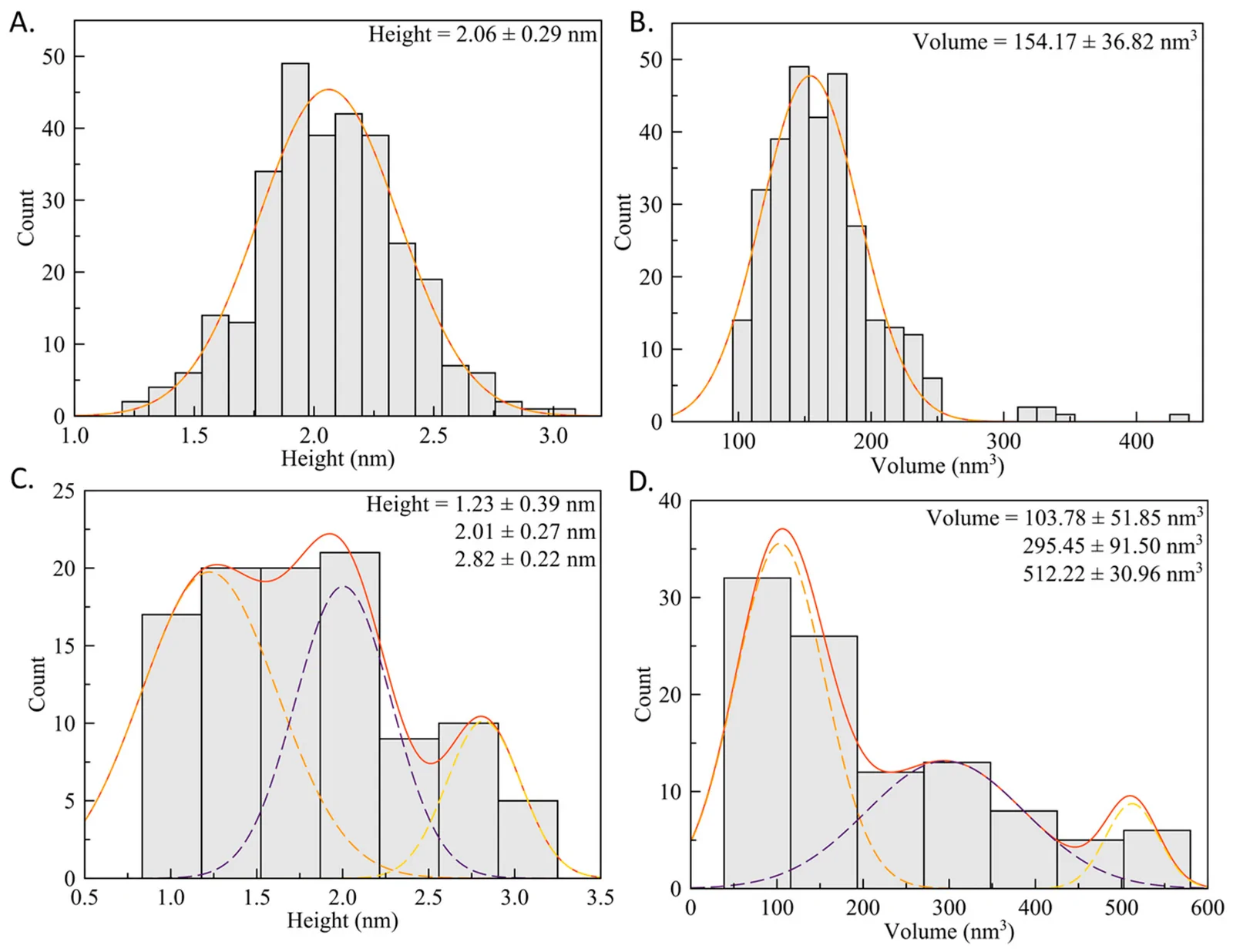
Height and volume distributions of wtSSB particles measured by AFM, without (**A**,**B**) and with (**C**,**D**) RecO. Data were fitted to one- and three-population (dotted lines) Gaussian distributions. Standard deviation was calculated as σ = dx/√(2·ln2). Data are reported as mean ± SD. The R^2^ for all the fits was ≥ 0.95.

**Figure A3:**
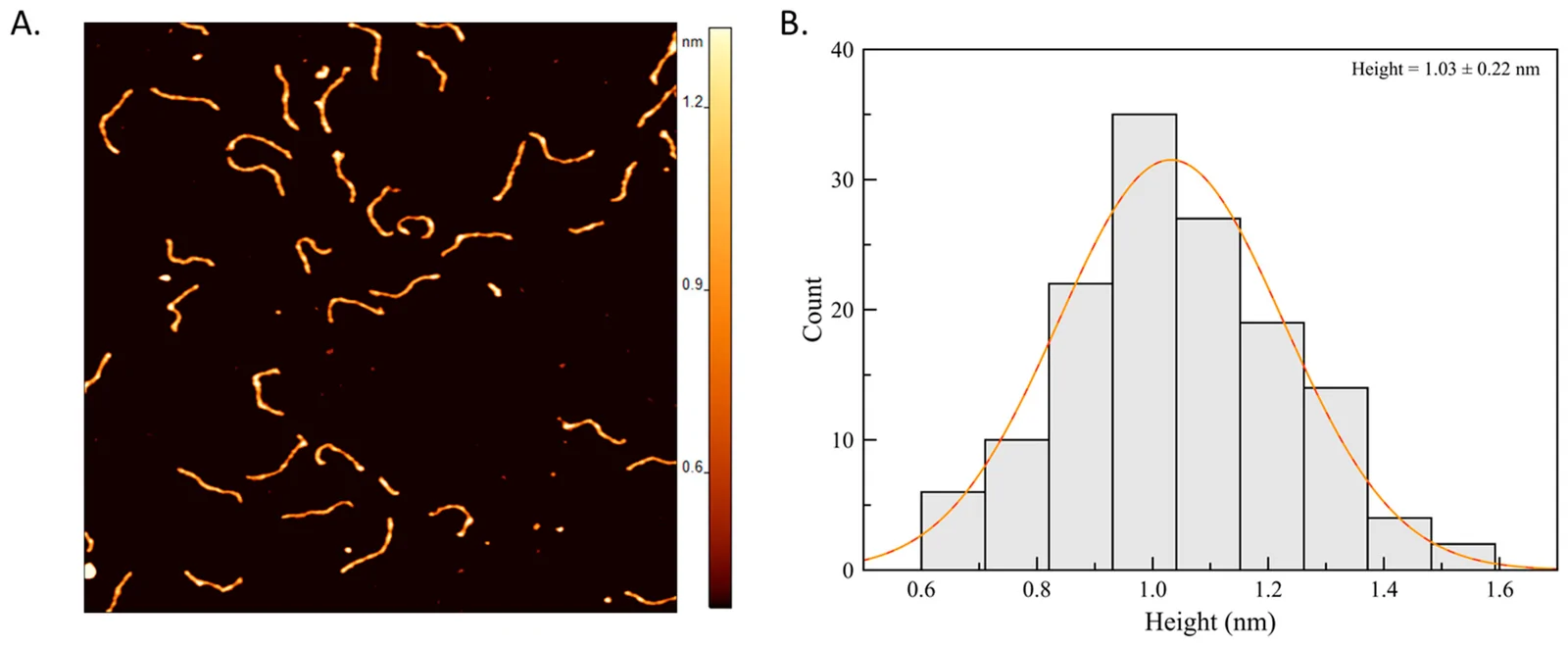
(**A**) AFM image of DNA substrate alone in the absence of any protein (SSB/variants or RecO). (**B**) Height distribution of dsDNA marker (internal control). Data were fit to a one-population Gaussian distribution. Standard deviation was calculated as σ = dx/√(2·ln2). Data are reported as mean ± SD. The R^2^ of the fit was ≥0.95.

**Figure A4:**
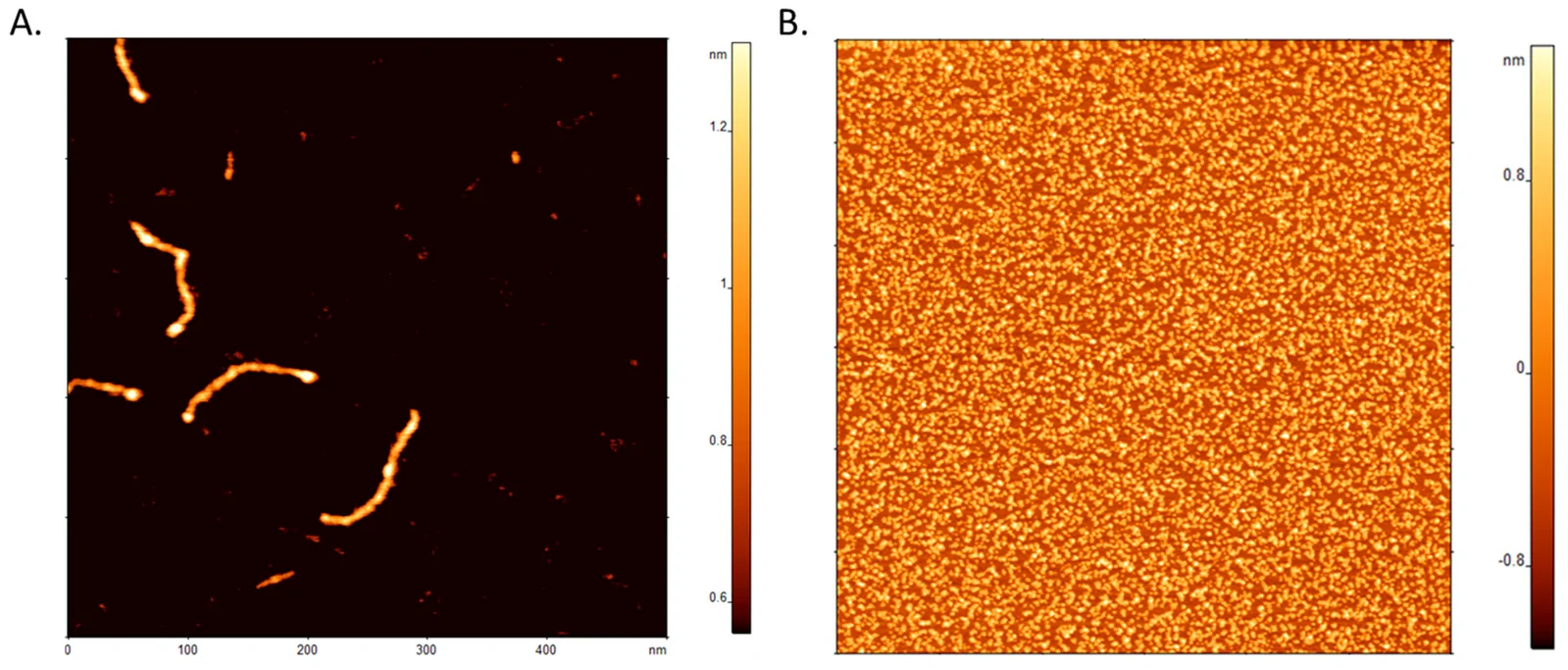
AFM imaging of RecO on the DNA substrate. (**A**) at 1:1—RecO to substrate ratio and (**B**) at 10:1—RecO to substrate ratio.

The DNA substrate for AFM imaging provides a controlled single-site ssDNA platform for binding of SSB and SSB variants. The substrate consists of a 389 bp double-stranded DNA (dsDNA) body bearing a 68-nucleotide single-stranded tail at one end. The dsDNA body was designed to serve two complementary purposes. First, it acts as a visual marker: its defined, uniform contour under AFM makes it immediately distinguishable from protein-bound or aggregated species, so that substrate molecules can be unambiguously identified in every image. Second, because a dsDNA segment of this length has a well-defined, reproducible height under AFM, it also functions as an internal standard within every field of view. Measuring the dsDNA height in each experiment and confirming that it remains constant across sessions and samples verifies AFM-tip and instrument performance, establishing internal consistency between experiments before any biological signal is interpreted. To this marker, we appended a randomized, non-complementary 68-nucleotide ssDNA segment at one end, providing a single, spatially defined binding site for SSB and its variants. To validate the substrate, we first imaged it in the absence of any protein. AFM images showed the expected morphology: a rod-like dsDNA body with a flexible single-stranded extension at one end, which may or may not be visible on the AFM image (Figure A3). The addition of wtSSB to the substrate resulted in the appearance of a discrete globular feature co-localized at one end of the rod-like structure, the ssDNA tail, confirming specific binding. Non-specific binding of SSB to the dsDNA regions of the substrate was avoided by maintaining an ionic strength of 50 mM and by adding Mg^2+^ ions to the buffer [33]. In the absence of RecO, 66.7% of ssDNA tails were occupied by wtSSB (*N* ≥ 321 occupied molecules; Figure 3A and Figure 4A,B, and Table A1), with protein-free dsDNA body regions in all cases, confirming that SSB binding is specific for the single-stranded region. Occupied complexes displayed a mean height of 2.06 ± 0.29 nm and a volume of 154.17 ± 36.82 nm^3^, consistent with values reported for single SSB tetramers bound to ssDNA in prior AFM studies [33,34]. These measurements validate the substrate and establish baseline parameters for the RecO binding and displacement experiments. Additionally, AFM imaging of RecO in the presence of a DNA substrate was performed at two different protein-to-substrate ratios. At 1:1 (150 nM each), distinct end binding between RecO and ssDNA was rarely observed, which was attributed to RecO’s 4-fold smaller size than SSB and to its lower affinity for ssDNA. At 10:1 (1.5 μM RecO to 150 nM ssDNA), the mica surface was non-specifically coated with RecO, which precluded quantification of morphological features such as height and volume (Figure A4).

### 2.5. RecO Efficiently Displaces wtSSB from the ssDNA Tail

Throughout this manuscript, we use “displacement” and “dissociation” interchangeably to refer to the same observable: loss of an AFM-visible SSB/variant complex from the ssDNA tail following RecO addition. Each AFM experiment was performed at least twice independently, and the results were pooled for statistical analysis. Replicate-level statistics are presented in Table A3 and Table A4. To test whether RecO can dissociate wtSSB from the ssDNA substrate, pre-formed wtSSB-ssDNA complexes were incubated with RecO and imaged by AFM. A striking reduction in ssDNA occupancy was observed upon RecO addition: the fraction of tails bearing SSB complexes decreased from 66.7% to 29.0% (*N* ≥ 102 occupied molecules; Figure 3B and Figure 4C,D; Table A1), representing an approximately 2.3-fold reduction relative to the RecO-free condition. Fisher’s exact test: *p* = 2.5 × 10^−28^, indicating a highly significant displacement (Table A2). In the presence of RecO, the majority of ssDNA tails appeared completely protein-free, with no alteration in substrate morphology (Figure 3B). However, the residual 29% protein-DNA complexes were found to be highly heterogeneous and split into three sub-populations with heights of 1.23 ± 0.39 nm, 2.01 ± 0.27 nm, and 2.82 ± 0.22 nm. The volume distributions were also highly heterogeneous and were divided into three distinct populations, with values ranging from approximately 103 to 512 nm^3^ (Table A1). The complexes that do remain bound could reflect several non-exclusive scenarios: molecules captured at different stages of the multi-step dissociation process, a subpopulation not yet engaged by RecO under these incubation conditions, or stable co-localized RecO/SSB complexes. Since the images are end-point rather than real-time recordings, our data cannot distinguish between these scenarios. Nevertheless, the predominance of fully cleared tails indicates that RecO efficiently removes SSBs from the defined ssDNA substrate.

### 2.6. Deletion of the Acidic Tip Abolishes RecO-Mediated Displacement: ΔC8

**Figure 5:**
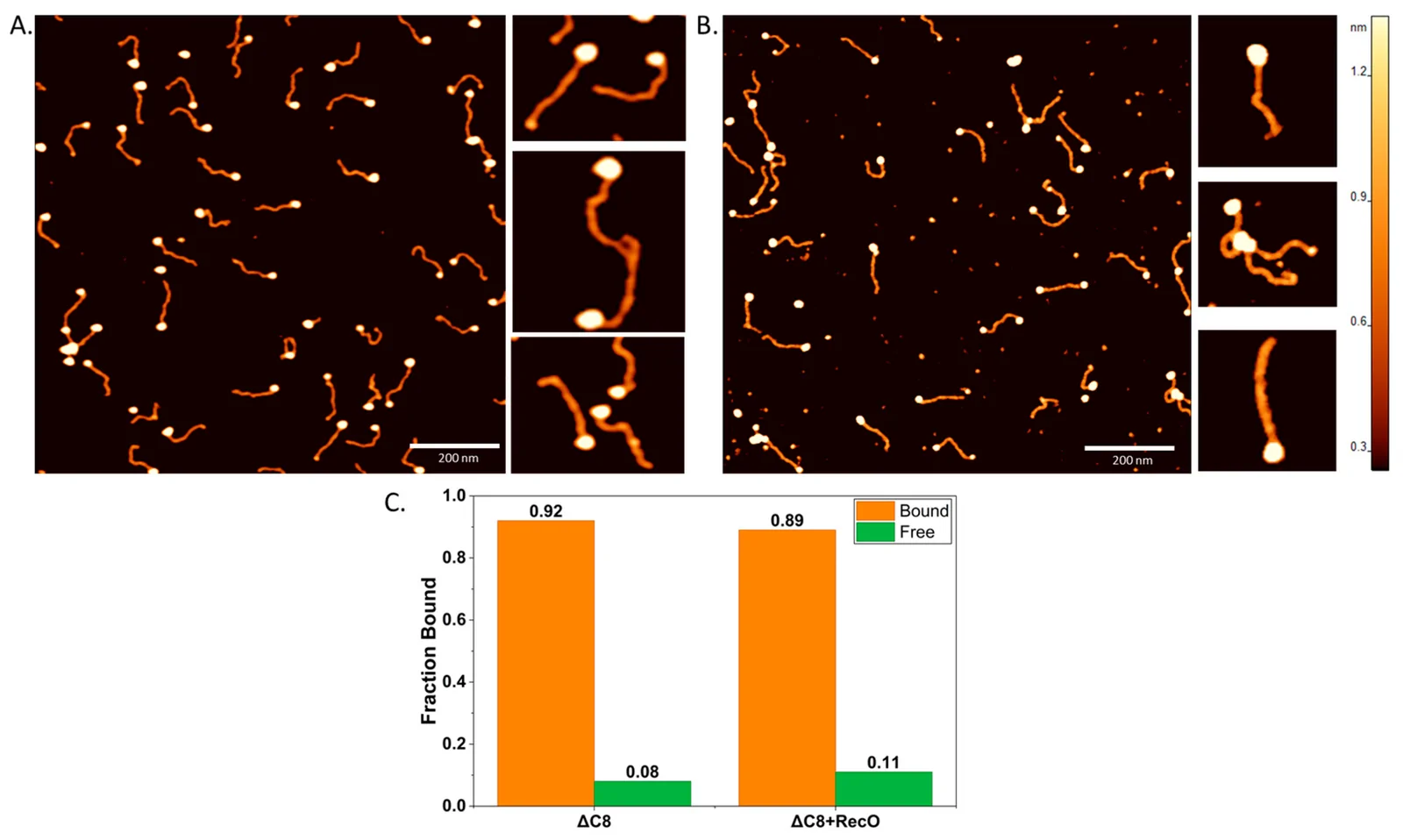
AFM imaging of ΔC8 on the DNA substrate ± RecO. (**A**) Representative field and zoomed-in insets, DNA + ΔC8 alone. (**B**) Representative field and zoomed-in insets, DNA + ΔC8 + RecO. Scale bars: 200 nm. (**C**) Fraction of DNA molecules scored as SSB-bound vs. free, ΔC8 alone vs. ΔC8 + RecO.

To determine whether the C-terminal acidic tip is required for RecO-driven displacement, we examined the ΔC8 variant, which lacks the tip entirely. In the absence of RecO, 92% of ssDNA tails were occupied by ΔC8 and formed protein complexes (*N* ≥ 543 occupied molecules; Figure 5A; Table A1), confirming that ΔC8 is fully competent to bind ssDNA. Strikingly, the dimensions of ΔC8-ssDNA complexes differed substantially from those of wtSSB complexes.

**Figure 6:**
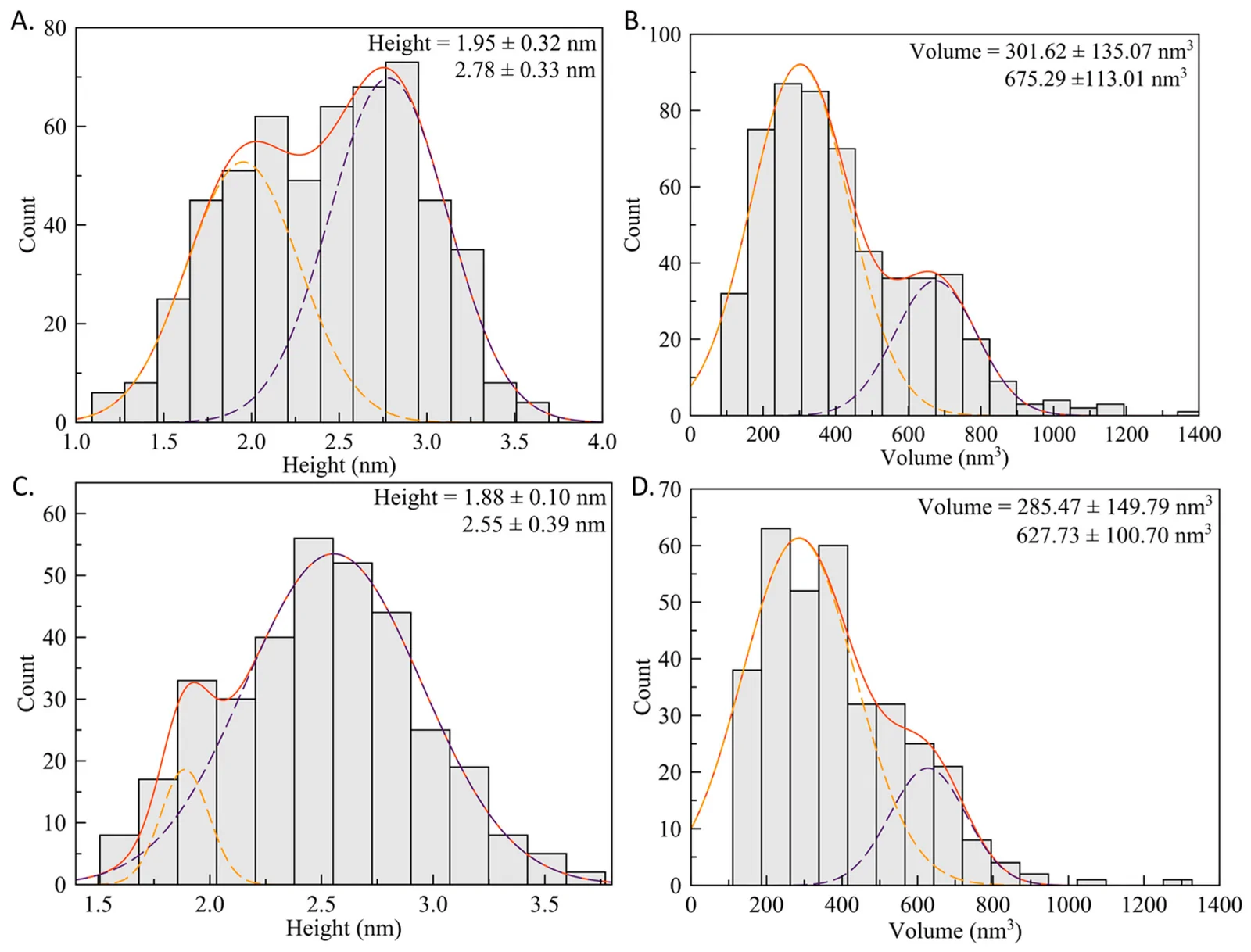
Height and volume distributions of ΔC8 particles measured by AFM, without (**A**,**B**) and with (**C**,**D**) RecO. Data were fit to a two-population (dashed lines) Gaussian distribution. Standard deviation was calculated as σ = dx/√(2·ln2). Data are reported as mean ± SD. The R^2^ for all the fits was ≥ 0.95.

The population distribution was bimodal, with mean complex heights of 1.95 ± 0.32 nm and 2.78 ± 0.33 nm (compared to 2.06 ± 0.29 nm for wtSSB), and apparent volumes of 301.62 ± 135.07 nm^3^ and 675.29 ± 113.01 nm^3^ respectively (Figure 6A,B). The subpopulation with smaller height and volume may be comparable to wild type, whereas the subpopulation with greater height has a volume approximately four times that of wtSSB complexes. We hypothesize that these altered dimensions reflect a shift in the wrapping mode equilibrium toward the less compactly wrapped (SSB)*_35_* configuration [35] or the assembly of multiple SSB tetramers on the 68 nt tail, either of which would produce complexes with elevated apparent volume when imaged by AFM.

Upon addition of RecO to ΔC8-loaded substrates, the ssDNA occupancy remained essentially unchanged: 89% of tails remained occupied (*N* ≥ 339 occupied molecules; Figure 5B; Figure 6C,D; Table A1), Fisher’s exact test; *p* = 0.072 (Table A2), indicating no significant change and closely matching the RecO-free condition. The volumes also remain comparable to the values in the absence of RecO (Figure 6C,D; Table A1). Within the sensitivity of this assay, RecO does not produce a detectable change in the ΔC8′s occupancy, height, or volume.

### 2.7. A Single Amino Acid Addition to the Tip Abolishes Displacement: A179

**Figure 7:**
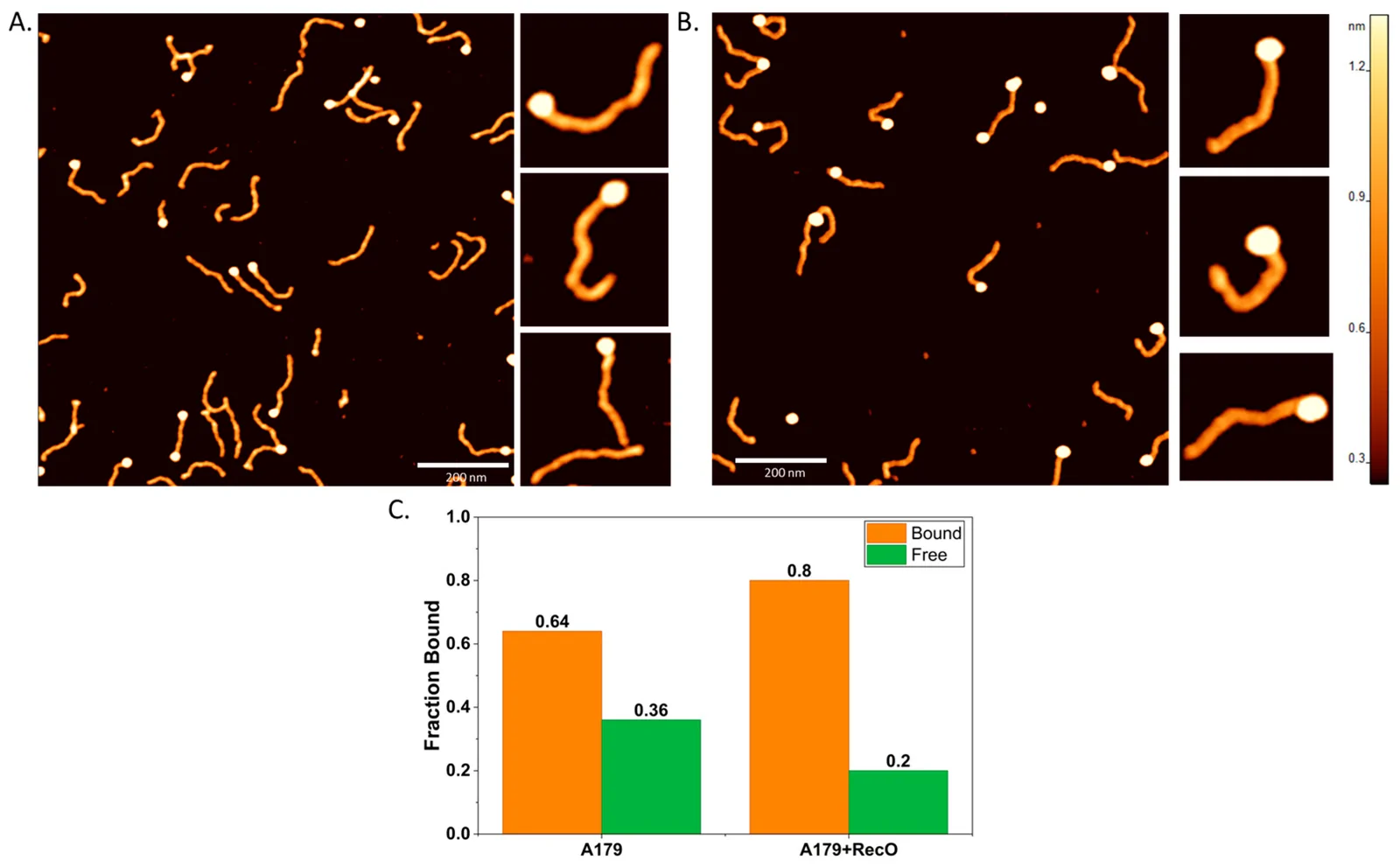
AFM imaging of A179 on the DNA substrate ± RecO. (**A**) Representative field and zoomed-in insets, DNA + A179 alone. (**B**) Representative field and zoomed-in insets, DNA + A179 + RecO. Scale bars, 200 nm. (**C**) Fraction of DNA molecules scored as SSB-bound vs. free, A179 alone vs. A179 + RecO.

To test the role of the tip’s sequence specificity in RecO engagement, we examined A179, which carries a single alanine appended to the C-terminus of SSB. In the absence of RecO, 64% of ssDNA tails were occupied by A179 (*N* ≥ 241 occupied molecules; Figure 7A; Table A1), confirming competent ssDNA binding.

**Figure 8:**
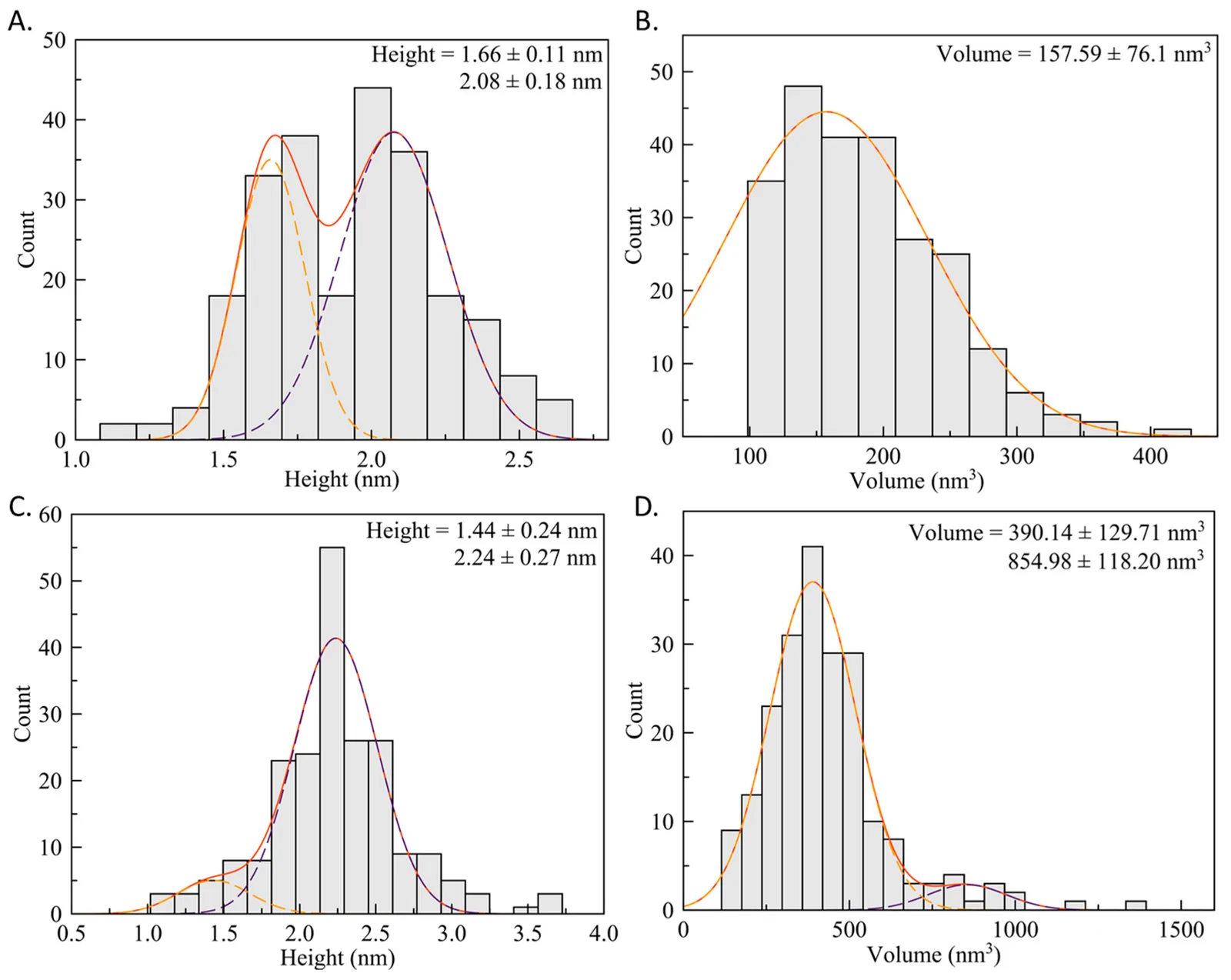
Height and volume distributions of A179 particles measured by AFM, without (**A**,**B**) and with (**C**,**D**) RecO. Data were fit to a one- or two-population (dashed lines) Gaussian distribution. Standard deviation was calculated as σ = dx/√(2·ln2). Data are reported as mean ± SD. The R^2^ for all the fits was ≥ 0.95, except for the height plots with and without RecO, where R^2^ ≥ 0.85.

The height of the protein yielded a bimodal distribution, in contrast to that of the wtSSB, and the volume distribution was unimodal and comparable to the wild-type conditions (Figure 8A,B and Table A1).

It should be noted that the R^2^ values for height distributions, both in the presence and absence of RecO, were ≥ 0.85 under Freedman–Diaconis (FD) binning. Deviating from the FD rule and adjusting the bin size yielded a unimodal distribution in the absence of RecO and improved the goodness of fit to > 0.95 in the presence of RecO. However, to maintain a uniform binning method, we retained FD binning and used the resulting lower R^2^ fit for comparisons. The modestly lower baseline occupancy and the change in height distribution compared to wtSSB may reflect a subtle effect of the mutation on ssDNA affinity or binding mode.

Interestingly, in contrast to wtSSB, the addition of RecO increased A179′s ssDNA occupancy to 80% (Fisher’s exact *p* = 1.5 × 10^−5^), indicating a statistically significant increase in occupancy (*N* ≥ 211 occupied molecules; Figure 7B and Figure 8C,D; Table A1). A single amino acid addition at the end of the tip is therefore sufficient to affect RecO-mediated displacement, although the mechanism behind these effects remains unclear. In addition, this variant accumulated more protein at the ssDNA end. The magnitude of the A179 volume increase (roughly 2.5- to 5-fold) initially raised the possibility of non-specific aggregation of RecO, A179, or both as an explanation that is at least as plausible as the formation of a stable, specific, non-functional co-complex. The molecular dynamics simulations presented below resolve this ambiguity in favor of a specific, structured interface. Both ΔC8 and A179 converge on the same functional outcome, no displacement, through two independent tip perturbations, indicating a genuine requirement for a correctly configured C-terminal tip.

### 2.8. Molecular Dynamics Simulations Reveal a Structural Basis for A179′s Non-Productive RecO Engagement

**Figure 9:**
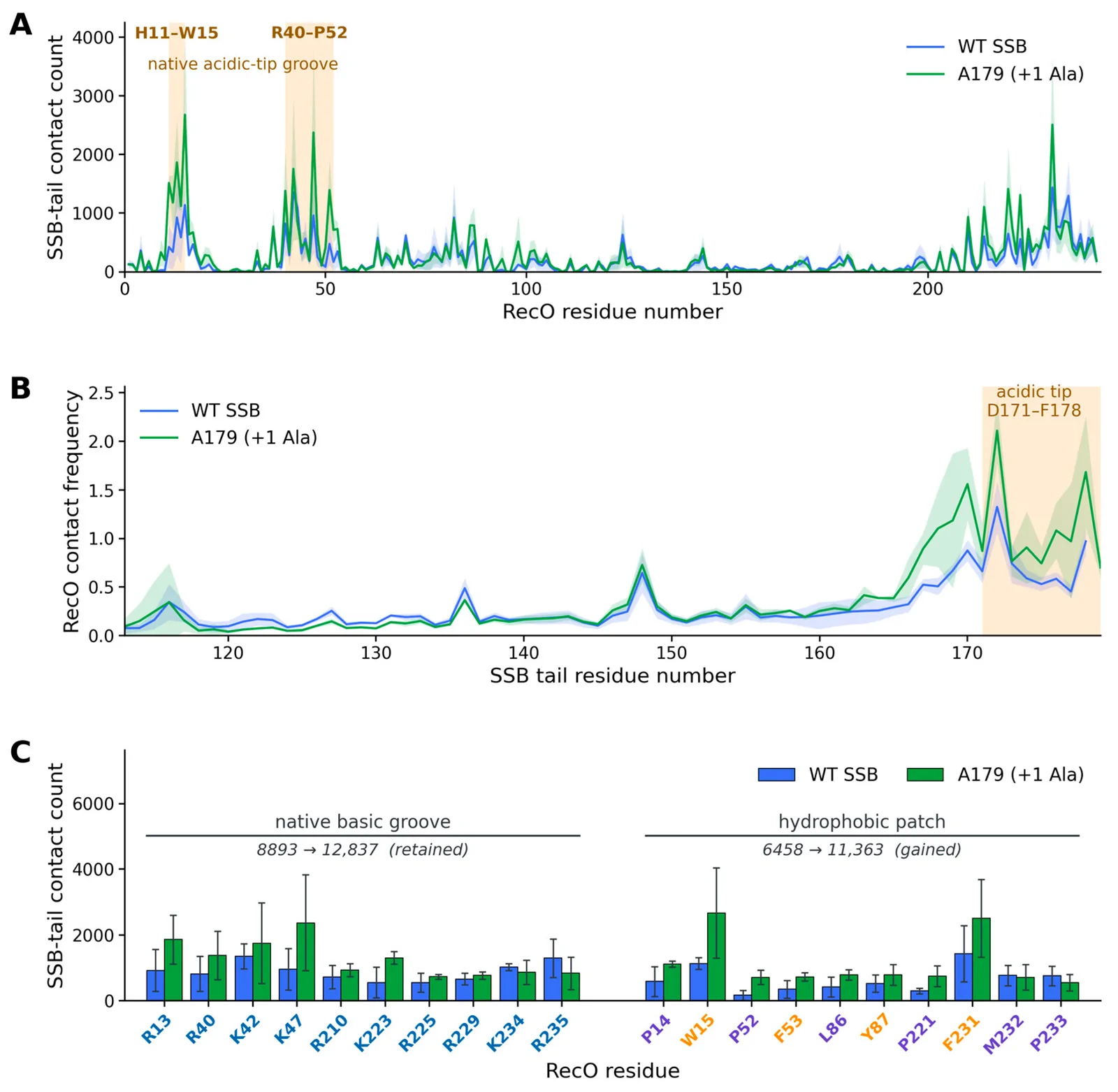
Molecular dynamics simulations of the RecO–SSB interaction for wtSSB and A179 (n = 3 replicates per variant). (**A**) RecO surface engaged by the SSB tail: per-residue contact counts across all 242 RecO residues, wtSSB (blue) vs. A179 (green); lines show the mean and shaded bands show the SD across replicates. Shaded regions mark the native acidic-tip groove (H11–W15 and R40–P52). (**B**) SSB tail residues (110–179) contacting RecO are plotted as contact frequency (mean ± SD, shaded bands); the acidic tip (residues 171–178) is shaded, and the A179 trace extends to the appended alanine at position 179. (**C**) SSB-tail contact counts for individual RecO residues of the native basic groove (R13, R40, K42, K47, R210, K223, R225, R229, K234, R235) and the hydrophobic patch (P14, W15, P52, F53, L86, Y87, P221, F231, M232, P233), wtSSB vs. A179; bars show mean ± SD. Summed counts for each group are given above the bars (wtSSB → A179).

The outcome of RecO’s engagement with SSB is determined by their protein–protein interface rather than by ssDNA binding per se. We performed coarse-grained molecular dynamics simulations of the RecO–SSB interaction for wtSSB and A179 (n = 3 independent replicates per variant; see Materials and Methods). We analyzed these trajectories from two complementary vantage points: which residues on RecO’s surface are contacted by the SSB tail (Figure 9A), and which residues along the SSB tail make those contacts (Figure 9B).

Figure 9A maps contact frequency across all 242 RecO residues, comparing wtSSB (blue) with A179 (purple), with shaded bands showing the spread across the three trajectories. The marked basic-groove residues (R210, R225, R229, K234) correspond closely to the positively charged rim of the crystallographically defined SSB-Ct pocket (Ryzhikov et al., PDB 3Q8D: Arg-210, Arg-225, Arg-229, Lys-206, Arg-203, [11], Figure 1), and engagement here tracks closely between the two variants. The marked hydrophobic-patch residues span a wider range (W15, F53, V66, L86, L90); of these, L86 and L90 lie immediately adjacent to the hydrophobic residues that line the floor of that same crystallographic pocket (Ile-84, Tyr-91), while W15, F53, and V66 fall well outside it, in RecO’s N-terminal OB-fold domain. A179 rises above wild type across the whole patch, most conspicuously at W15, where the peak roughly doubles, indicating both deeper engagement of the authentic pocket floor and genuinely novel contacts further from it.

Figure 9C collapses Panels A–B into the two functional groups. Engagement of the basic-groove rim is statistically indistinguishable between the variants (wtSSB 2965 ± 559 vs. A179 3307 ± 173). Engagement of the hydrophobic-patch group roughly doubles in A179 overall (wtSSB 2295 ± 497 vs. A179 5009 ± 849), driven mainly by the F172–W15 aromatic contact, the single strongest A179 pair, reproduced in two of the three runs, and located outside the known crystallographic pocket. The contribution from L86/L90, immediately adjacent to the pocket’s native hydrophobic floor, cannot be separated from deeper engagement of the authentic site at this level of grouping and would need a per-residue (rather than per-group) comparison to resolve.

Taken together, the panels show that appending a single alanine at position 179 does not cost A179 the native RecO basic-groove rim. Instead, the tail’s extra length is accommodated by increased engagement of the hydrophobic region in and around the same pocket: part of this (L86, L90) may reflect deeper penetration of the authentic hydrophobic floor (Ile-84, Tyr-91), while part, most notably W15, lies well outside the known pocket, in RecO’s N-terminal OB-fold domain, and represents a genuinely novel contact. Rather than a clean switch from a specific native interface to an entirely separate non-native one, the data are more consistent with a single interface that becomes both deeper and more extensive in A179, retained at its native rim, but larger and less well-registered around its hydrophobic core. This provides a structural rationale for the AFM observation of an increase in the volume of A179 in the presence of RecO.

## 3. Discussion

This study provides the first direct nanoscale evidence for SSB displacement by the RecO protein in *E. coli.* Using single-molecule AFM imaging, we demonstrate (Table A1 and Table A2) that the addition of RecO results in a 2.3-fold reduction in ssDNA occupancy (Table A1 and Table A2). The displacement of SSB by RecO is consistent with the biological role of RecO in clearing ssDNA gaps for RecA loading in the RecFOR pathway [13,36,37].

The displacement of the SSB from the ssDNA end cannot be explained by direct competition between SSB and RecO for ssDNA, since RecO’s intrinsic affinity for ssDNA is substantially lower than that of SSB [13,22,23]. Instead, the determining factor is the specific interprotein interaction between RecO and SSB, specifically involving the C-terminal tip. Our variant data localizes this interaction with striking precision: ΔC8, which lacks the terminal tip, shows no RecO-induced displacement, whereas A179, which retains the full-length tip but carries a single additional residue beyond the native C-terminal Pro-Phe (P-F) terminus, is equally resistant to RecO-induced displacement. This minimal change completely abolished the displacement activity, suggesting that RecO may recognize the SSB C-terminal tail through structurally precise, sequence-specific docking interactions. Our observation is consistent with the RecO–SSB-Ct co-crystal structure, in which terminal tip residues insert into a defined pocket on RecO’s surface [11].

Upon RecO addition, the residual bound population of wtSSB (Table A1 and Table A2) resolved into a trimodal distribution (a series of intermediates) (103, 295, and 512 nm^3^) rather than a single alternative state. One plausible explanation for this heterogeneity and for the increase in volume is that displacement may proceed through intermediates rather than a direct, single-step release (Table A1). This heterogeneity could be associated with SSB transitioning from a fully wrapped (SSB)_65_-like state (mean volume 154 nm^3^) toward the more extended (SSB)_35_ mode during productive displacement; changes in volume during this transition have been reported previously [34]. Alternatively, SSB tetramer dissociation and the formation of a transient RecO–SSB complex could be another plausible explanation. By contrast, ΔC8 exhibits substantially altered baseline volumes and heights relative to wtSSB (Table A1 and Table A2), reflecting a ~4-fold increase in volume. RecO addition does not alter the ΔC8 volume distribution, suggesting that RecO fails to establish productive contacts with this truncated variant.

By contrast, A179 remains essentially wtSSB-like at baseline (unimodal volume, comparable occupancy), but becomes bimodal and enlarged upon RecO addition, indicating that its altered architecture is induced specifically by the non-productive RecO interaction.

These results establish that RecO actively removes SSB–doing so only when the SSB C-terminal tip is intact and correctly positioned. This resolves a long-standing tension between the static, peptide-bound crystal structure of the SSB–RecO interface and the dynamic requirement, central to the RecFOR pathway, that SSB ultimately vacate the ssDNA it protects.

The MD simulation of RecO–SSB in the absence of ssDNA, at the tail interface, revealed that engagement of the basic-groove rim residues of the crystallographically defined SSB-C terminal binding pocket (RecO—R210, R225, R229, K234) is statistically indistinguishable between wtSSB and A179 [11]. The added alanine allows an extended tail engagement with RecO, which is accommodated by an increased engagement of the hydrophobic region in and around the pocket, establishing additional non-native contacts, which is possibly one reason for the lack of displacement observed in the AFM experiments.

Taken together, ΔC8 and A179 represent opposite failure modes of the same interface: ΔC8 is a loss-of-interaction variant (no change in occupancy, height, or volume in the presence of RecO), in which the contact cannot form, whereas A179 is a gain-of-interaction variant, in which additional, non-native contacts enlarge the interface. Only the wild-type interface, complete but not expanded beyond its native boundaries, supports productive displacement, underscoring that the specificity of this protein–protein contact, not merely its strength, governs the outcome.

This mechanism is consistent with the findings of Inoue et al., that the SSB-Ct peptide alone decreases SSB’s ssDNA binding affinity [21]. Furthermore, single-molecule studies in *Deinococcus radiodurans* show that RecO efficiently displaces SSB from ssDNA without consuming ATP, despite SSB binding ~300-fold more tightly than RecO [25]. The strict C-terminal tip dependence of SSB displacement in our AFM experiments directly supports this model.

A closer look at the two variants indicates which property, altered ssDNA binding or altered RecO engagement, underlies the loss of displacement in each case. For A179 the two can be separated, and the responsible factor is the RecO interaction. A179 binds ssDNA essentially as wtSSB does (64.1% versus 66.7% occupancy, with comparable complex volumes). Yet, RecO clearly engages it, raising occupancy to 79.9% and increasing complex volume several-fold. Correspondingly, the simulations show that this engagement retains the native basic-groove contacts while roughly doubling contact with RecO’s hydrophobic patch (Figure 9). A179 therefore binds ssDNA normally but docks RecO through a shifted, non-productive interface. For ΔC8 the two factors cannot be separated, because the C-terminal tip is bifunctional: it associates with the ssDNA-binding face of the OB-fold, lowering ssDNA affinity [21], while also serving as the recognition element for RecO. Its deletion therefore frees the ssDNA-binding face and removes the RecO handle in a single step, consistent with the elevated ΔC8 occupancy (92.0%) and with the weaker RecO binding measured by SPR. We note that our simulations contain protein only, with no ssDNA present, so this interpretation is a hypothesis rather than a result.

We hypothesize that the SSB C-terminal tip serves a physiological role beyond enabling RecO recognition: it functions as a fidelity checkpoint [26,32,38]. SSB coats ssDNA throughout the genome—including at replication forks, resected double-strand breaks, and repair intermediates—where its protective coating must be preserved. By requiring the C-terminal tip to terminate at its native sequence, the cell restricts productive engagement to a single, precisely registered interface, so that SSB is released only where RecO has bound correctly. In the cell, this regulation is also aided by partner proteins such as RecF and RecR, and how their presence affects ssDNA clearance is indeed very interesting and remains an important question for future work. Different bacteria have thus evolved distinct molecular solutions to the same functional requirement, clearing SSB at the right place and time, with *E. coli* exploiting protein–protein interaction specificity. Our observation of this preference for specificity over affinity may be enabling RecO’s regulatory role in vivo.

## 4. Materials and Methods

### 4.1. Protein Expressions and Purification

All SSB variants (wtSSB, ΔC8, and A179) were expressed in *Escherichia coli* and purified according to established protocols from the Bianco laboratory [39,40]. RecO was purified using a strategy specifically designed to address its tendency to accumulate in the insoluble fraction upon overexpression. A construct encoding RecO with an N-terminal His-tag and SUMO solubility tag was co-expressed with SSB in *E. coli* to improve the partitioning of RecO into the soluble fraction. Following detergent-assisted cell lysis and clarification by centrifugation, the soluble lysate was applied to a Ni-Sepharose affinity column. The bound His-tagged material was eluted and dialyzed against high-salt buffer (600 mM NaCl) in the presence of His-tagged SenP2 protease to cleave the SUMO tag. The digest was reloaded onto a Ni-Sepharose column; His-tagged components (uncleaved fusion, His-SenP2, free His-SUMO) were retained, and the flow-through containing untagged RecO was collected. RecO was further purified on a Mono S cation-exchange column, where it bound strongly and was separated from co-purifying SSB by a low-salt wash. RecO was eluted with a linear NaCl gradient (200 mM to 1 M NaCl over 10 column volumes) followed by a 2 M NaCl wash to recover tightly bound fractions. Pure RecO fractions were pooled, dialyzed into storage buffer (20 mM Tris-HCl pH 7.5, 200 mM NaCl, 1 mM DTT, 60% *v*/*v* glycerol), and stored at −80 °C. 60% glycerol was specifically chosen based on the available literature for RecO purification and storage [41]. Purity was confirmed by SDS-PAGE, and protein concentration was determined by absorbance at 280 nm. All proteins retained their native amino acid sequence composition, and our purification strategies did not introduce any additional amino acids into the sequence.

### 4.2. DNA Substrate Preparation

Double-stranded DNA (dsDNA) was prepared as previously described [42,43] and digested with SapI. The digested dsDNA was purified using a Qiagen (Hilden, DE, USA) gel extraction kit and quantified by a NanoDrop spectrophotometer (ND-1000, Thermo Fisher, Waltham, MA, USA). To generate the single-stranded DNA (ssDNA) tail, a random 91-nucleotide oligonucleotide (5′-/5Phos/GCT CCA GAC CGA GTC GTA ATC TTT AAC ATA GTT AGC TCT TCA GAA TTA TCC CTA AGA ACG ACC TGA ACA CTA ATA GTG TAT TCC ATA CTT T-3′) was annealed to a 20-nucleotide adapter (5′-AAG ATT ACG ACT CGG TCT GG-3′) by rapid heating to 95 °C followed by gradual cooling to 4 °C overnight. The adapter was designed to leave three nucleotides at the 5′ end of the 91 nt oligonucleotide that were complementary to the SapI-digested dsDNA segment. The annealed oligonucleotides were then hybridized to the SapI-digested dsDNA by increasing the temperature to 37 °C and gradually cooling to 16 °C. The dsDNA:ssDNA tail molar ratio during annealing was maintained at 1:10. T4 DNA ligase and T4 ligation buffer were added to ligate the annealed DNA constructs, and the mixture was incubated at 16 °C overnight. The ligated DNA was separated by 2% agarose gel electrophoresis, and the band of interest was excised and purified using a Qiagen gel extraction kit. The purified DNA was quantified by a NanoDrop spectrophotometer. Both oligonucleotides were ordered from Integrated DNA Technologies (IDT, Coralville, IA 52241, USA).

### 4.3. Surface Plasmon Resonance

SPR experiments were performed on an OpenSPR instrument (Nicoya Lifesciences, Kitchener, ON, Canada) using streptavidin/biotin/streptavidin sensor chips prepared according to the manufacturer’s instructions. Streptavidin was first immobilized on the sensor surface at 0.5 μM; 200 nM biotinylated oligonucleotide was then captured on the streptavidin layer to present an ssDNA substrate. SSB/variants (200 nM) were flowed over the oligo-coated chip to achieve stable immobilization of SSB on the ssDNA, and unbound SSB was removed by washing to a stable baseline. RecO was subsequently injected as the analyte at a series of concentrations to monitor its interaction with the surface-tethered SSB-ssDNA complex. Association and dissociation phases were recorded, and sensorgrams were analyzed using TraceDrawer (version 1.10.1) software. Due to limitations in fitting quality and reproducibility inherent to RecO aggregation, quantitative kinetic parameters could not be reliably extracted; SPR data are discussed only as a qualitative indication of the effect of acidic tip deletion on RecO binding.

### 4.4. AFM Sample Preparation and Imaging

All experiments were performed in AFM buffer: 10 mM Tris-HCl (pH 7.4), 50 mM NaCl, 10 mM MgCl_2_, and 1 mM DTT (DTT added freshly before each experiment) [33]. DNA substrate and SSB/variant were mixed at a 1:2 molar ratio (1 uL of each stock) and incubated for 10 min at room temperature to form SSB-DNA complexes. For RecO displacement assays, 1 uL of RecO was added to the preformed SSB/variant-DNA mixture to achieve a final 1:2:2 molar ratio (DNA:SSB/variant:RecO) and incubated at room temperature for an additional 10 min. The reaction was diluted with AFM buffer to a final DNA concentration of 2 nM. SSB concentration is reported as the tetramer concentration. DNA substrate was used at a concentration of 150 nM, SSB was maintained at 300 nM, and RecO at 300 nM to achieve the molar ratios mentioned above.

In total, 50 μL of the diluted sample was deposited onto freshly cleaved bare mica and allowed to adsorb for 2 min. The surface was rinsed thoroughly with Milli-Q water to remove unbound material, then dried under a gentle stream of nitrogen gas. AFM images were acquired in tapping mode in air using a MultiMode AFM/NanoScope IIId system using TESPA probes (Bruker Nano Inc, Camarillo, CA, USA) equipped with TESP-V2 probes (nominal spring constant 42 N/m, resonance frequency ~320 kHz). Scan areas of 3 × 3 μm were collected at 1536 × 1536-pixel resolution at a 1.0 Hz scan rate. Images were processed in FemtoScan Analysis software (Advance Technologies Center, Moscow, Russia) with first-order line-by-line flattening only; no additional filtering was applied. Multiple independent experiments, each comprising an independent incubation/deposition reaction, AFM sample preparation, and imaging session, were carried out for each condition. RecO and the DNA substrate were each drawn at random from two independently prepared batches across these replicates, and each SSB protein (wtSSB and each variant) was purified as a single batch used throughout.

### 4.5. AFM Data Analysis

Protein-DNA complexes were identified by the presence of a globular feature co-localized with the ssDNA tail of the substrate. ssDNA occupancy was calculated as the fraction of substrate molecules carrying a bound complex on the ssDNA tail. Differences in ssDNA occupancy (the percentage of tails bound by a protein complex) between the RecO-free and RecO-added conditions were assessed for each SSB variant using a two-tailed Fisher’s exact test applied to the underlying 2 × 2 contingency table of occupied and unoccupied molecule counts. Fisher’s exact test was used rather than a chi-square approximation because it computes an exact *p*-value from the hypergeometric distribution rather than relying on a large-sample approximation. A threshold of *p* < 0.05 was used to define statistical significance. Because each condition comprised multiple independent AFM experiments, occupancy was additionally assessed at the level of individual replicates to confirm that the result was not driven by pooling molecules across experiments. For each SSB variant, the percentage of occupied molecules was calculated separately for each independent replicate, and these replicate-level percentages were compared between RecO-free and RecO-added conditions using the Mann–Whitney U test. As a complementary parametric approach, replicate-level percentages were arcsine-square-root transformed and compared using Welch’s *t*-test, which does not assume equal variance between groups. Both replicate-level tests were consistent with the pooled molecule-level Fisher’s exact test for each variant.

Complex height and apparent volume were measured using the particle-analysis module of FemtoScan Analysis. For statistical particle analysis, the grain size distributions were visualized as histograms with bin widths determined by the Freedman–Diaconis rule [44] (h = 2 × IQR × n ^−1/3^), where h is the bin size, IQR is the interquartile range of the data, and n is the sample size. This rule is robust to outliers and does not assume normality. Histograms were then constructed using this calculated bin width, and Gaussian models were fit to the resulting distributions using MagicPlot v2.8.1 to extract peak parameters including mean grain size, standard deviation (σ), and relative peak area. Bin size sensitivity was systematically tested for all samples to confirm that Gaussian model parameters remained stable across reasonable binning ranges; exceptions to this robustness are indicated where they occur.

### 4.6. Coarse-Grained Molecular Dynamics Simulations

Full-length *E. coli* SSB was modeled by combining the crystallographic OB-fold core (PDB 1QVC) [45] with the disordered C-terminal region (residues 113–178) from the AlphaFold [46] Model of UniProt P0AGE0, assembled as the physiological homotetramer; *E. coli* RecO (242 residues) was modeled likewise. Two constructs were simulated: wild type and A179. Systems were coarse-grained with martinize2 using the MARTINI 3 force field [47,48], with an elastic network on the folded OB-fold core only, solvated with explicit MARTINI water and counter-ions in a 24.6 nm cubic box, and propagated with GROMACS 2025.3 [49]. SSB–RecO trajectories of 25 microseconds each were produced (triplicates for wild type and A179), together with one 25-microsecond trajectory per variant of the SSB tetramer alone. Contacts were scored at a 0.6 nm bead–bead cut-off, and a frame was counted as bound when the minimum SSB–RecO distance fell below this threshold.

## Data Availability

The original contributions presented in this study are included in the article. Further inquiries can be directed to the corresponding author.

## Abbreviations

The following abbreviations are used in this manuscript:

AFM	Atomic Force Microscopy
SSB	Single-Stranded DNA-Binding Protein
RecO	Recombination Protein O
RecA	Recombinase Protein A
SD	Standard Deviation
dsDNA	Double-Stranded Deoxyribonucleic acid
ssDNA	Single-Stranded Deoxyribonucleic acid
SPR	Surface Plasmon Resonance
ITC	Isothermal Titration Calorimetry
IDL	Intrinsically Disordered Linker
OB-fold	Oligonucleotide/oligosaccharide binding-fold
FD	Freedman–Diaconis (binning rule)
SIP(s)	SSB-interacting protein(s)
PDB	Protein Data Bank
MD	Molecular dynamics
SEM	Standard error of mean
SSB-Ct	SSB C-terminal peptide
IQR	Interquartile range
KD	Dissociation constant
Nt/bp	Nucleotides/base-pair(s)

## Appendix A

**Table A1:** AFM height and volume measurements across SSB variants with and without RecO. Values are mean ± S.D.

Protein	N	Gaussian Distribution	%SSB/Variant Bound	Height (nm)	Volume (nm^3^)	% Population Split (Based on Height Traces)
wtSSB	481	Unimodal	67	2.06 ± 0.29	154.17 ± 36.82	NA

wtSSB + RecO	361	Trimodal	29	2.01 ± 0.27	295.45 ± 91.50	34.5
				2.82 ± 0.22	512 ± 30.96	14.4
A179	376	Bimodal for height	64	1.66 ± 0.11	157.59 ± 76.1	63.2
		Unimodal for volume		2.08 ± 0.18		36.8
A179+RecO	264	Bimodal	80	1.44 ± 0.24	390.14 ± 129.71	9.6
				2.24 ± 0.27	854.98 ± 118.20	90.4
ΔC8	590	Bimodal	92	1.95 ± 0.32	301.32 ± 135.07	42.1
				2.78 ± 0.33	675.29 ± 113.01	57.9
ΔC8 + RecO	383	Bimodal	89	1.88 ± 0.10	285.47 ± 149.79	8.7
				2.55 ± 0.39	627.73 ± 100.70	91.3

**Table A2:** Statistical comparison of ssDNA occupancy by SSB with and without RecO. Occupied/total molecule counts for each SSB variant, before and after RecO addition, and the corresponding two-tailed Fisher’s exact test *p* value. *p* < 0.05 was considered statistically significant.

Protein	(−)RecO	(+)RecO	Fold Change (−RecO/+RecO)	Odds Ratio	Fisher’s *p*	Observation
wtSSB	321/481 = 66.7%	102/358 = 28.5%	2.3	5.04	2.5 × 10^−28^	Significant
A179	241/376 = 64.1%	211/264 = 79.9%	0.80	0.45	1.46 × 10^−5^	Significant
		
ΔC8	543/590 = 92.0%	339/383 = 88.5%	1.04	1.50	0.072	Not Significant
		

**Table A3:** Replicate level DNA occupancy.

Protein	Condition	Replicate	Occupied	Unoccupied	Total	% Occupied
wtSSB	−RecO	1	111	25	136	81.6
	−RecO	2	75	36	111	67.6
	−RecO	3	58	25	83	69.9
	−RecO	4	77	74	151	51
	−RecO	Pooled	321	160	481	66.7
wtSSB	+RecO	1	52	95	147	35.4
	+RecO	2	15	87	102	14.7
	+RecO	3	35	74	109	32.1
	+RecO	Pooled	102	256	358	28.5
ΔC8	−RecO	1	285	21	306	93.1
	−RecO	2	258	26	284	90.8
	−RecO	Pooled	543	47	590	92
ΔC8	+RecO	1	117	10	127	92.1
	+RecO	2	110	19	129	85.3
	+RecO	3	112	15	127	88.2
	+RecO	Pooled	339	44	383	88.5
A179	−RecO	1	141	83	224	62.9
	−RecO	2	100	52	152	65.8
	−RecO	Pooled	241	135	376	64.1
A179	+RecO	1	111	29	140	79.3
	+RecO	2	100	24	124	80.6
	+RecO	Pooled	211	53	264	79.9

**Table A4:** Statistical tests of the RecO effect on occupancy, molecule-level vs. replicate-level.

Protein	Test	Level	Statistics	*p* Value	Odds Ratio	Conclusion
wtSSB	Fisher’s exact test	Pooled (481 vs. 358)	-	2.5 × 10^−28^	5.04	Significant
wtSSB	Mann–Whitney U	Replicates (4 vs. 3)	U = 12.0	0.057	-	Complete separation (*p*-value floor for n)
wtSSB	Welch’s *t*-test (arcsine)	Replicates (4 vs. 3)	t = 4.16	0.011	-	Significant
ΔC8	Fisher’s exact test	Pooled (590 vs. 383)	-	0.072	1.50	Not significant
ΔC8	Mann–Whitney U	Replicates (2 vs. 3)	U = 5.0	0.40	-	Not significant
ΔC8	Welch’s *t*-test (arcsine)	Replicates (2 vs. 3)	t = 1.49	0.234	-	Not significant
A179	Fisher’s exact test	Pooled (376 vs. 264)	-	1.5 × 10^−5^	0.45	Significant
A179	Mann–Whitney U	Replicates (2 vs. 2)	U = 0.0	0.333	-	Complete separation (*p*-value floor for n)
A179	Welch’s *t*-test (arcsine)	Replicates (2 vs. 2)	t = −10.27	0.019	-	Significant
